# Proteomic characterization of persisters in *Enterococcus faecium*

**DOI:** 10.1186/s12866-023-03162-8

**Published:** 2024-01-03

**Authors:** Charlotte Le Pont, Benoît Bernay, Mattéo Gérard, Anne Dhalluin, François Gravey, Jean-Christophe Giard

**Affiliations:** 1 UNICAEN, Univ Rouen Normandie, INSERM, DYNAMICURE UMR 1311, Caen, F-14000 France; 2 Plateforme Proteogen SFR ICORE 4206, Université de Caen Normandie, Caen, 14000 France; 3 Department of infectious agents, UNICAEN, Univ Rouen Normandie, INSERM, DYNAMICURE UMR 1311, CHU Caen, Caen, F-14000 France

**Keywords:** *Enterococcus faecium*, Persister cells, Proteomic, Stress response

## Abstract

**Background:**

*Enterococcus faecium* is a Gram-positive bacterium, naturally present in the human intestinal microbiota, but is also an opportunistic pathogen responsible for healthcare-associated infections. Persisters are individuals of a subpopulation able to survive by arrest of growth coping with conditions that are lethal for the rest of the population. These persistent cells can grow again when the stress disappears from their environment and can cause relapses.

**Results:**

In this study, we highlighted that ciprofloxacin (10-fold the MIC) led to the formation of persister cells of *E*. *faecium*. The kill curve was typically biphasic with an initial drop of survival (more than 2 orders of magnitude reduction) followed by a constant bacterial count. Growth curves and antimicrobial susceptibility tests of these persisters were similar to those of the original cells. In addition, by genomic analyses, we confirmed that the persisters were genotypically identical to the wild type. Comparative proteomic analysis revealed that 56 proteins have significantly different abundances in persisters compared to cells harvested before the addition of stressing agent. Most of them were related to energetic metabolisms, some polypeptides were involved in transcription regulation, and seven were stress proteins like CspA, PrsA, ClpX and particularly enzymes linked to the oxidative stress response.

**Conclusions:**

This work provided evidences that the pathogen *E. faecium* was able to enter a state of persister that may have an impact in chronic infections and relapses. Moreover, putative key effectors of this phenotypical behavior were identified by proteomic approach.

**Supplementary Information:**

The online version contains supplementary material available at 10.1186/s12866-023-03162-8.

## Background

As part of the intestinal microbiota, enterococci are opportunistic pathogens causing various health care infections such as sepsis, urinary or abdominal infections especially in immunocompromised people. These Enterococci are also able to form biofilms on the catheters and have a pathogenicity island that promotes initial colonization [1, 2]. The species most frequently found are *Enterococcus faecalis* and *Enterococcus faecium* [3]. *E. faecalis* appears to be the most virulent while *E. faecium* poses more therapeutic problems related to antibiotic resistance mostly beta-lactams due to mutations within the penicillin binding protein (PBP) type 5. In addition, vancomycin (VRE) resistant strains have emerged since the 1980s [4]. *E. faecium* is part of the ESKAPE group of pathogens (for *E. faecium*, *Staphylococcus aureus*, *Klebsiella pneumoniae*, *Acinetobacter baumannii*, *Pseudomonas aeruginosa* and *Enterobacter* spp.) which includes pathogenic bacteria highlighted by the medical association “Infectious Diseases Society of America” recognized as virulent and resistant to antibiotics [5].

In parallel with the major health care challenge that is the emergence of multi-resistant bacteria, the ability of certain bacteria to enter a state of persistence poses the problem of recurrent and chronic infections. Persister cells are defined as a subpopulation with the same susceptibility as the reference strain, and whose individuals are able to survive durably by growth arrest, during exposure to conditions lethal to the rest of the population. This resistance is not due to specific genes but appears as a phenotypic adaptation [6]. Thus, non-persisters die very quickly while persisters remain alive, without dividing nor growing. This is a transient mechanism and the progeny also give rise to a mixture of susceptible bacteria and persister cells. However, regrowth can be observed when the antibiotic and/or the stress disappear from the environment.

Various stress conditions involved in the formation of persisters have been discovered recently, mainly in Gram-negative bacteria. In *Escherichia coli* (the most studied bacterium) it has been demonstrated that, in front of a nitrogen deficiency, the number of persisters surviving treatment with ciprofloxacin increased [7]. The authors also showed that this starvation leads to a stringent response which is at the origin of the formation of persister cells. The role of the SOS system in the formation of persisters was shown in *E. coli* [8]. Indeed, strains mutated in this system were less able to form persisters in presence of ciprofloxacin, an antibiotic known to cause DNA breaks. Salicylate has also been shown to cause persister cells formation via the production of ROS (Reactive Oxygen Species) in *E. coli* [9]. These ROS can cause a mitigation of membrane potential as well as a decrease in metabolism that are two parameters promoting the formation of persisters in *E. coli*. Moreover, in vivo, it seems that the combination of various stress parameters leads to the formation of persisters, as is the case in *Salmonella* when it is internalized by macrophages [10, 11]. In addition, toxin-antitoxin systems are involved in persister cells formation in *E. coli* [12]. In *E. faecalis*, the level of the alarmone pp(G)pp involved in the stringent response, was likewise required for antimicrobials tolerance [13].

The clinical impact of these persister cells is now well established because they escape antibiotic treatments and can be the cause of chronic infections [14]. A study conducted in 2016 revealed that bacteria from the ESKAPE group were able to form persisters in the presence of antibiotics [15]. However, very little data is currently available on persisters of *E. faecium*, a major opportunistic pathogen. The objective of our study was therefore to provide knowledge on persistent *E. faecium* following antibiotic stress. In order to identify the molecular mechanisms triggered in this bacterial subpopulation, global proteomic studies were carried out.

## Results

### Ciprofloxacin as a persister-inducible stress in ***E. faecium***

**Fig. 1:**
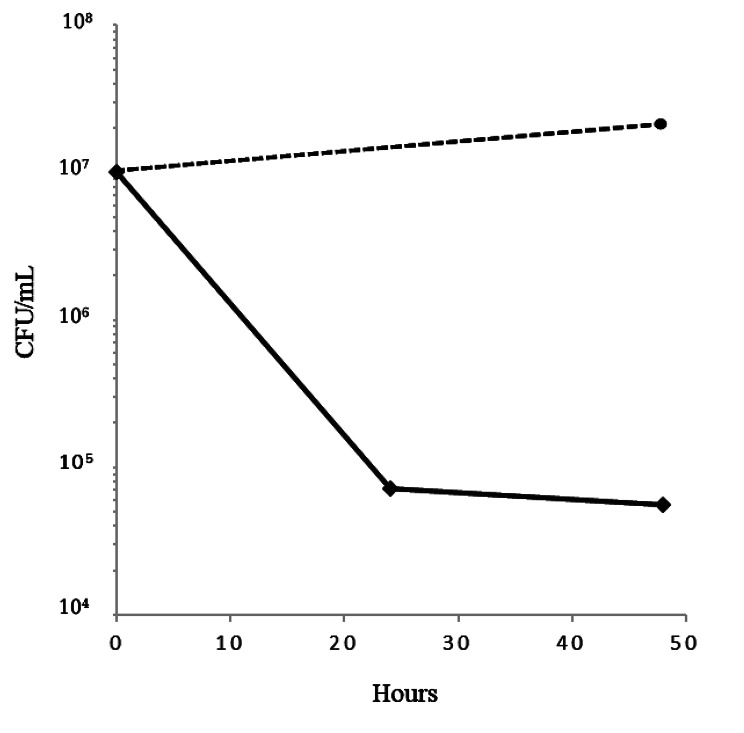
Representative biphasic survival curve of three biological replicates of *E. faecium* AUS0004 in the presence of 10-fold the MIC of ciprofloxacin (black line) and without ciprofloxacin (dotted line). The antibiotic was added to a growing cell culture (OD_600_ of 0.3)

The minimum inhibitory concentration (MIC) of ciprofloxacin for the AUS004 strain of *E. faecium* was 2 µg/mL. We evaluated the survival of this susceptible bacterium in the presence of 10-fold the MIC (Fig. 1). Cells exposed to this high concentration of ciprofloxacin at the exponential phase (OD_600_ of 0.3) died rapidly during the first 24 h with a loss of viability of more than two orders of magnitude. Then, the number of countable bacteria remained almost constant for another 24 h. This type of biphasic killing curve corresponded to a characteristic evolution of survival which results in the formation of persister cells (Fig. 1). Three colonies that survived 48 h in the presence of ciprofloxacin were therefore subcultured for further analyses.

### Phenotypical and genomic characterization of persister cells of ***E. faecium***

Antibiograms of persister strains collected after treatment with ciprofloxacin (10-fold MIC) were performed to ensure that the colonies recovered following the challenge had not survived thanks to the acquisition of resistance mechanisms. As shown in Table 1, the antimicrobial resistance profiles of the persisters were identical to that of our reference. Etest® were also carried out in order to verify whether the MICs of ciprofloxacin were modified. The results revealed that these MICs for the persisters were unchanged (of 2 µg/mL).

**Table 1:** Antibiograms of the original strain of *E. faecium* AUS0004 and three strains of persisters harvested after 48 h in presence of 20 µg/mL of ciprofloxacin (10 x MIC).

Antibiotics (µg)^a^	Diameters of inhibition (mm)			
	AUS0004	Strain 1	Strain 2	Strain 3
AMP (2)	**6** ^**b**^	**6**	**6**	**6**
IMP (10)	**6**	**6**	**6**	**6**
NXM (10)	23	24	23	23
RIF (5)	**12**	**14**	**14**	**13**
ERY (10)	**6**	**6**	**6**	**6**
CMN (2)	**6**	**6**	**6**	**6**
QDF (15)	**19**	**21**	**20**	**20**
TGC (15)	32	31	31	32
LIN (10)	30	32	30	30
LVX (5)	22	22	21	21
GME (30)	19	19	18	19
HLS (300)	23	23	22	23
VNC (5)	**15**	**14**	**14**	**14**
TEC (30)	20	21	21	20
NFE (100)	15	15	15	15
FOS (200)	25	25	23	26

^a^ The acronyms of the antibiotics correspond to: ampicillin (AMP), imipenem (IMP), norfloxacin (NXM), rifampicin (RIF), erythromycin (ERY), clindamycin (CMN), quinuspristin-dalfopristin (QDF), tigecycline (TGC), linezolid (LIN), levofloxacin (LVX), gentamicin (GME), streptomycin (HLS), vancomycin (VNC), teicoplanin (TEC), nitrofurantoin (NFE), fosfomycin (FOS).

^b^ Values written in bold indicated a clinical categorization of “Resistant”

Growth monitoring was also performed for the reference strain and for the persisters to verify that the development of the bacterial cells was not impacted. Thus, the growth kinetics in MH medium of all the persister cells analyzed were identical (Fig. S1).

To determine whether the persistent character of the selected strains was linked to a genetic determinant, the whole genome sequencing of the original strain and of a persister strain after treatment with ciprofloxacin was undertaken. We compared these genomic sequences and highlighted that they were identical. We identified ten areas of uncertainty in the sequences. We therefore designed PCR primers allowing the amplification of the fragments surrounding these alterations which were sequenced by the Sanger technology. Our data confirmed the identity of the sequences (data not shown). These results revealed that the persistence of *E. faecium* was linked to phenotypic adaptation and not to genomic modification.

### Proteomic analysis of persister cells of ***E. faecium***

For the proteomic study, proteins of *E. faecium* were extracted from cells harvested at the middle of exponential phase (OD_600_ of 0.3) (Expo), after 48 h of stationary phase (T_48h_), and after 48 h in presence of 10-fold MIC of ciprofloxacin (starting from an OD_600_ of 0.3) (T_48hcip_). Note that, based on the published protocol [16], T_48h_ and T_48cip_ cells were initially treated with magnetic beads coupled with propidium iodide to limit the presence of altered or dead cells. For each sample, about 1400 of the 2826 proteins potentially coded by the chromosome have been identified and quantified using protein extracts from three biological replicates. We then compared the different profiles of the cells from the exponential and stationary phase (T_48h_ versus Expo), as well as before and after treatment with ciprofloxacin (T_48hcip_ versus Expo). The selection criteria for determining that a protein was differentially present were a Log_2_FC (Fold-Change) less than − 1.5 or greater than 1.5 associated with a corrected p-value less than 0.05. With regard to the high number of proteins identified, these values were chosen in order to focus on the proteins whose abundances were most obviously and statistically modified.

**Fig. 2:**
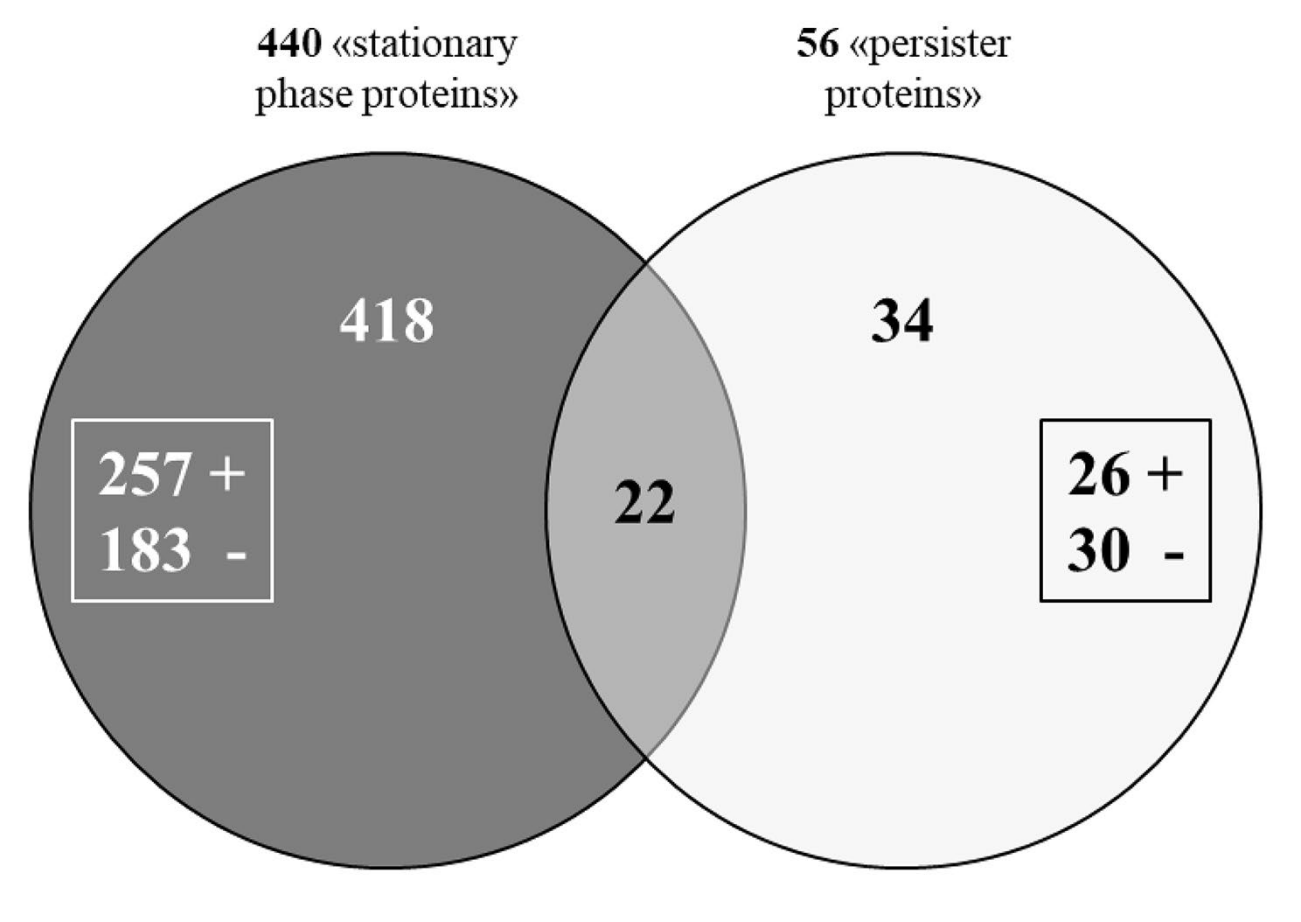
Venn diagram of stationary phase and persister proteins. In the dark circle are indicated stationary phase proteins (comparison T_48h_ vs. Expo) and in the light circle the persister proteins (comparison T_48hcip_ vs. Expo). + and - indicate the number of significantly more and less abundant proteins, respectively, in stationary phase and persister cells

440 stationary phase proteins (T_48h_ versus Expo) were found significantly more (n = 257) or less abundant (n = 183) (Fig. 2, Table S1). On the other side, 56 proteins were differentially abundant when compared the profiles from growing cells and persisters (T_48h_cip versus Expo) (Fig. 2; Table 2). Of these, 26 were overproduced (Log_2_FC between 1.5 and 3.8) while 30 were underrepresented (Log_2_FC between − 1.5 and − 8.2). As shown in Figs. 2 22 corresponded to stationary phase proteins and 34 were specific to persisters (16 present in lower quantity and 18 more abundant). It is also interesting to note that three stationary phase proteins appeared to be regulated in an opposite way in the persisters. Thus, CspA, a protein involved in the stress response, appeared less present in stationary phase cells (the most underrepresented), whereas it was more abundant in the persister ones (Table 2, Table S1). PrsA, a protein playing a major role in the extracellular folding of several secreted proteins, was also more present in the persisters whereas it was less present in the stationary phase cells. Finally, a hydrolase from the haloacid dehalogenase superfamily (HAD – enzyme with both beta-phosphoglucomutase and hydrolase activities) was more present in bacteria harvested in stationary phase but appeared less abundant in persisters.

**Table 2:** Characteristics of the 56 proteins differentially abundant when compared the proteomic profiles from persisters and growing cells (T48hcip vs. Expo)

Gene	Protein annotation (Uniprot)	Protein description	Log_2_ FC T_48hcip_ vs. Expo^a^	Log_2_ FC T_48h_ vs. Expo^b^
11,191	I3U1C7	M15B subfamily muramoylpentapeptide carboxypeptidase	-8.20	-5.82
10,503	I3U1A5	Peptidoglycan-binding protein	-6.80	-3.53
11,429	Q3XXV0	Amino acid ABC superfamily ATP binding cassette transporter, binding protein	-5.44	-4.33
*mtlD*	Q3Y1G8	Mannitol-1-phosphate 5-dehydrogenase	-4.48	
*galP*	Q3Y292	MFS transporter, SP family galactose:H + symporter	-4.35	
*sagA*	I3U595	Secreted antigen A	-4.32	-2.17
11,462	Q3Y0G5	Uncharacterized protein	-4.00	
12,555	Q3Y185	ErfK/YbiS/YcfS/YnhG family protein	-3.66	-4.78
*ppiA*	Q3XZ44	Foldase protein PrsA	-3.66	1.83
*rseP*	I3U2R6	Zinc metalloprotease	-3.50	
10,635	Q3Y0A7	Uncharacterized protein	-3.48	
*cysK*	Q3XX46	Cysteine synthase	-3.46	
10,888	Q3Y2R0	Flavodoxin	-3.36	-1.56
10,870	Q3Y2P3	Bacteriophage minor structural protein	-3.30	
*mdoB*	Q3XZ26	Phosphatidylglycerol–membrane-oligosaccharide glycerophosphotransferase	-2.94	
*murA*	Q3XY92	UDP-N-acetylglucosamine 1-carboxyvinyltransferase	-2.84	-1.72
11,465	Q3Y0G2	Protein of hypothetical function DUF984	-2.80	
*rpmG*	I3U4T5	50 S ribosomal protein L33	-2.48	-2.94
*rpsN*	Q3XYX6	30 S ribosomal protein S14 type Z	-2.26	
*mvk*	Q3XZL3	Mevalonate kinase	-2.24	
10,382	I3TZ18	NADH:flavin oxidoreductase/NADH oxidase	-2.09	-2.02
10,028	Q3XYZ5	Haloacid dehalogenase (HAD) superfamily hydrolase	-1.92	-2.50
p*yrD2*	I3U217	Dihydroorotate oxidase (fumarate)	-1.90	
12,850	Q3Y0Z2	Uncharacterized protein	-1.82	
*clpX*	Q3XZ71	ATP-dependent Clp protease ATP-binding subunit ClpX	-1.82	
12,761	I3U5U7	Uncharacterized protein	-1.78	
11,804	Q3Y303	MarR family transcriptional regulator	-1.72	
*pgsA*	Q3Y0P3	CDP-diacylglycerol–glycerol-3-phosphate 3-phosphatidyltransferase	-1.69	
*murB*	Q3Y1W9	UDP-N-acetylenolpyruvoylglucosamine reductase	-1.67	
*mobC*	I3U5U5	Mobilization protein C	-1.57	-3.45
12,063	I3U3U9	M20/M25/M40 family peptidase	1.51	
*polA*	I3U2E8	DNA polymerase I	1.51	
*erpQ*	Q3XZG3	ErpQ protein	1.54	
*cspA*	I3U0N2	Cold shock protein Csp	1.57	-6.12
*pdp*	Q3XXR6	Pyrimidine-nucleoside phosphorylase	1.59	
*accA*	Q3Y0R8	Acetyl-coenzyme A carboxylase carboxyl transferase subunit alpha	1.68	2.74
*glpF*	Q3XYL4	Glycerol MIP family major intrinsic protein channel protein	1.74	2.59
10,691	Q3Y1 × 6	Flavin reductase	1.78	
*papS*	I3U153	Poly A polymerase	1.96	
*uxuA*	Q3XZP4	Mannonate dehydratase	1.97	
*ahpC*	Q3XZP1	Alkyl hydroperoxide reductase C	2.02	
*dacA*	I3U3E0	Serine-type D-Ala-D-Ala carboxypeptidase	2.05	4.42
*panE*	Q3Y316	2-dehydropantoate 2-reductase	2.06	
*dps*	I3U5I6	DNA-binding protein Dps	2.10	
*cad*	I3U5L5	Sex pheromone cAD1	2.24	
*rbsR*	I3TZ89	Ribose transcriptional regulator	2.33	
*mdlA*	Q3XXU7	Multidrug ABC superfamily ATP binding cassette transporter, ABC/membrane protein	2.35	1.70
12,664	I3U5K0	LacI family transcriptional regulator	2.53	2.55
*pphA*	I3U2F5	Phosphoprotein phosphatase	2.56	2.03
12,270	Q3XWI3	GNAT family acetyltransferase	2.78	
10,507	I3TZE3	GyrI-like domain-containing protein	2.79	
*qor*	I3TYH2	NADPH:quinone reductase	3.03	
11,264	I3U1K0	Uncharacterized protein	3.27	
*msrA4*	Q3XZS1	Peptide methionine sulfoxide reductase MsrA	3.65	2.17
*nusB*	Q3Y2G2	Transcription antitermination protein NusB	3.76	2.11
*tufA*;*tufA2*	Q3XX23	Elongation factor Tu	3.80	2.26

^a^ Fold change in abundance. Negative value indicates that the protein was less abundant in persisters than in growing cells.

^b^ Fold changes are indicated when the proteins were also identified as stationary phase proteins (T_48h_ vs. Expo).

The proteins differentially produced in persister cells were involved in various energy metabolisms and translation (Table 2). Moreover, four proteins were involved in transcriptional regulation and two proteins corresponded to ABC transporters. We also found the quorum-sensing protein cAD1 (peptide pheromone) and DNA repair-related polymerase I (PolA) to be more abundant in persisters. Interestingly, in addition to CspA and PrsA mentioned above, 5 other proteins were linked to the stress response. The protease ClpX, known to play a role in degradation of unfolded proteins was less abundant into persister cells. It is worth noting that four enzymes involved in the oxidative stress response of *E. faecium* have been identified in our proteomic study: the NADH oxidase (less abundant in persisters), the alkyl hydroperoxide reductase subunit C (AhpC), the DNA binding protein Dps and the methionine sulfoxide reductase (MsrA). These last three proteins were significantly over produced in the persisters (Table 2).

## Discussion

The objectives of this work were to determine whether ciprofloxacin treatment led to the formation of persisters in *E. faecium* and to identify the molecular mechanisms put in place during this process. These persisters were analyzed and, as expected showed no changes in their antibimicrobial resistance profiles, ciprofloxacin MICs and growth behaviors. After whole genome sequencing, we were also able to demonstrate that no genomic alterations were present in our persister strain. Michiels and collaborators have already shown that the LMG 8148 strain of *E. faecium* exhibited biphasic killing kinetics in the presence of gentamicin (400 µg/mL corresponding to 50-fold the MIC) indicating the formation of persisters but not with the quinolone levofloxacin (15-fold the MIC) [15]. It can be assumed that this lack of persistence may be due to the fact that the treatment was performed with stationary phase cells which were naturally more resistant at that time.

With persisters resulting from ciprofloxacin treatment, a proteomic study was carried out showing that 56 proteins were more or less abundant, of which 22 were stationary phase proteins. We also observed that the quorum-sensing protein cAD1 molecule was more abundant in the persisters. It has been shown that quorum-sensing signaling molecules promotes the formation of persisters in *P. aeruginosa* [17] whereas the quorum sensing-regulated toxins PSM (phenol-soluble modulin) reduced the *S. aureus* persister cells frequency upon ciprofloxacin treatment [18]. Further studies are needed to assess the role of pheromone synthesis in persistence status in *E. faecium*.

Furthermore, our proteomics data shed light on the importance of stress proteins in persister cells. Thereby, we found a cold-shock like protein which was strongly repressed during stationary phase whereas it was more abundant in persisters. In *E. coli*, the cold-shock like protein CspD, a member of CspA family, has been shown to be involved in the formation of persister cells [12]. These data suggest that the accumulation of CspA could play a role in the formation of persisters in *E. faecium*.

The PrsA protein (encoded by *ppiA* gene) was likewise more present in the persisters and less present in stationary phase cells. In *Bacillus subtilis*, a homologue of PrsA is known to be surface-exposed and involved in the protein secretion and the production of fully mature polypeptides [19]. Interestingly, this peptidylprolyl isomerase is also homologous to the EF0685 enzyme of *E. faecalis* important for NaCl resistance and virulence [20]. Recently, Willett and co-workers showed that PrsA of *E. faecalis* was indeed required for GelE (gelatinase) activity, which is a major virulence factor [21]. The ClpX protease, playing a key role in the quality control of proteins during ribosomal translation and in the degradation of unfolded proteins during stress response, was less abundant in persisters [22]. This may be correlated with the drastic drop of translational activity of the cells coping with the antimicrobial challenge.

Despite the addition of ciprofloxacin in our experiments which induced DNA strand breaks leading to a SOS response, the only persister’s protein linked to the DNA repair was the polymerase I enzyme. On the other hand, an oxidative stress response was clearly triggered in the implementation of persistence mechanisms. Thus, the alkyl hydroperoxide reductase subunit C (AhpC) responsible for the detoxification of reactive oxygen species (ROS) was present in higher amount in persisters. In addition, Dps that binds and protects DNA from oxidative damages, as well as MsrA (methionine sulfoxide reductase) which reduces the vulnerable amino acid methionine oxidized by ROS [23], were significantly more abundant into persisters. At the same time, NADH oxidase, which can play a role in the production of H_2_O_2_ [23], was less abundant than in untreated cells. All this contributes to dealing with ROS produced by bacterial cells or by the host during infection. The importance of the oxidative stress response in the formation of persister cells has been showed in both Gram positive and negative bacteria. In *S. aureus*, the *msaABCR* operon was involved in the persister cells formation and also regulated several genes required for the resistance against oxidative stress [24, 25]. During infection, *Streptococcus pneumoniae* cells were exposed to H_2_O_2_ resulting in an oxidative stress response and expression of ROS-detoxifying enzymes. This adaptative response triggered the formation of fluoroquinolone-persisters of pneumococci [26]. A similar scenario was recently observed in *Salmonella* where the host cell oxidative stress promoted antimicrobial persisters. Reactive nitrogen species (RNS) produced by macrophages locked bacteria in a persistent state through tricarboxylic acid (TCA) cycle intoxication [11]. Although several stress conditions are known to elicit persisters formation, it seems that in vivo, the oxidative stress corresponds to the main mechanism leading to this antibiotic recalcitrance state and may constitute an interesting target avoiding the treatment failure and the relapse of infection. In this context, analyzes of targeted mutant strains particularly affected in the response to oxidative stress (such as *ahpC*, *dps* or *msrA*) could be carried out to confirm their key roles in the formation of persisters and in survival to antimicrobial treatments.

## Conclusions

Our study revealed that a high concentration of ciprofloxacin promoted the formation of persister cells in *E. faecium*, an important opportunistic pathogen. Moreover, by global proteomic approach, several “persister-proteins” have been identified, among which stress proteins mainly linked to the oxidative stress response. Based on these data, it will therefore be important to adapt the treatments using pertinent antibiotics and concentrations, or by combining them with molecules which will make it possible to eradicate or prevent the formation of this recalcitrant bacterial subpopulation to avoid recurrent infections.

## Methods

### Bacterial strain and growth conditions

The strain AUS0004 of *E. faecium* was used for this study [27]. It has VanB-type resistance to vancomycin and was isolated in 1998, at Austin Hospital (Melbourne, Australia) from the blood of a patient (NCBI – GenBank: CP003351.1). BHI (Brain Heart Infusion) broth was used to cultivate *E. faecium* at 37° C without agitation. Plate counts were performed on TS (tryptone soya) agar plates incubated 48 h at 37 °C and MH medium (Mueller-Hinton) was used for antibiograms and cultures with antibiotics.

### Persisters assay

100 µL of the overnight culture of *E. faecium* were washed with MH medium and used to inoculate 9.9 mL of MH broth and incubated at 37 °C until an OD_600_ of 0.3 was reached. At this time point, ciprofloxacin was added to obtain 20 µg/mL corresponding to 10-fold the minimum inhibitory concentration (MIC). Samples were taken at 24 and 48 h to determine the number of CFUs (Colony Forming Units) by plate counts after serial dilutions.

### Determination of MICs and antibiograms

A bacterial suspension of density equivalent to a MacFarland of 0.5 was used to inoculate an MH agar plate using a sterile swab. An Etest® (Biomérieux, Marcy-l’Etoile, France) containing ciprofloxacin was loaded onto the agar and incubated overnight at 37 °C before MIC determination. In parallel, an antibiogram of Enterococci was performed by disk diffusion method using the following molecules (Bio-Rad, Hercules, CA, USA): ampicillin (2 µg), imipenem (10 µg), norfloxacin (10 µg), rifampicin (5 µg), erythromycin (10 µg), clindamycin (2 µg), quinuspristin-dalfopristin (15 µg), tigecycline (15 µg), linezolid (10 µg), levofloxacin (5 µg), gentamicin (30 µg), streptomycin (300 µg), vancomycin (5 µg), teicoplanin (30 µg), nitrofurantoin (100 µg), fosfomycin (200 µg). The interpretations were carried out according to the European Committee on Antimicrobial Susceptibility Testing (EUCAST) guidelines (https://www.eucast.org/clinical_breakpoints, 2022).

### Whole genome sequencing

To perform whole genome sequencing by the Illumina technique, an overnight culture was prepared with wild type strain *E. faecium* AUS0004, as well as with a persister strain harvested after 48 h in presence of ciprofloxacin (10 x MIC). From these cultures, DNA extractions were carried out using “NucleoBond® AXG 100 Columns” and “NucleoBond® Buffer Set III” buffers, according to the supplier’s recommendations (Macherey-Nagel, Hoerdt, France). A mechanical lysis step was added by transferring cell pellets into screw-capped tubes containing 500 µg of glass beads and disrupted using the Fast Prep instrument (MP Biomedical LLC, Santa Ana, CA, USA) for 3 min at 6.5 m/s. At the end of the protocol, in order to precipitate the DNAs, the solutions were left overnight at -20 °C after addition of isopropanol. The tubes were then centrifuged at 10,000 rpm for 25 min at 4 °C. The DNA pellets were collected and then washed with 70% ethanol. DNAs were dried, resuspended in 500 µL of water and quantified with Nanodrop (Thermo Fisher Scientific, Hampton, NH, USA). Finally, libraries were prepared using the DNA prep kit and sequenced with the Illumina Nextseq 500 sequencer by the Platform for Shared Microbiology (P2M) of the Pasteur International Bioresources Network (PIBNet; Institut Pasteur, Paris, France). After quality controls using fastqc (https://www.bioinformatics.babraham.ac.uk/projects/fastqc/), genomes were assembled using SPAdes v3.12 with recommended parameters for Illumina sequencing. We then compared the sequence of our original strain with that of the persistent strain in order to detect putative mutations using the snippy pipeline (https://github.com/tseemann/snippy).

### Protein extractions and mass spectrometry analysis

Cell pellets were obtained from 20 mL of cultures harvested at OD_600_ of 0.3, after 48 h of stationary phase and from persisters (48 h in presence of ciprofloxacin). In order to avoid extracting proteins from dead cells, we first used the protocol published by Sulaiman [16]. Briefly, the cells from 48-hour cultures or peristers were resuspended with 100 µL of saline buffer. Then, 100 µL of a suspension of magnetic beads (100 µg, 50 nm in diameter) (MACSflex MicroBead Kit, Miltenyi Biotec, Paris, France) coupled to propidium iodide (50 µg/mL) (Sigma-Aldrich, Saint-Louis, Missouri, USA) were added. The mixture was incubated for 15 min at room temperature, in the dark and then placed in a magnetic rack (MagAttract® Magnetic Rack, Qiagen, Hilden, Germany). The propidium iodide, linked to the beads, will interact with the DNA of cells whose membrane was altered, including dead cells. These magnetic beads were retained by the magnet, and thus only intact cells can be recovered. This step was repeated three times to remove as many beads as possible. The tubes containing the living cells were centrifuged at 6000 rpm, 15 min at 4 °C, and the pellets were resuspended with 500 µL of the recovery buffer (Tris HCl 50 mM, Na_2_SO_4_ 50 mM, glycerol 15%) and stored at -80 °C. For protein extractions, cells were disrupted by adding 500 µg of glass beads and using the Fast Prep instrument as previously described for DNA extractions. Protein quantifications were performed with the “Pierce BCA protein assay kit” (Thermo Fisher Scientific) according to the supplier’s recommendations.

For our global proteomic analysis by Mass Spectrometry (MS), 5 µg of each protein extract were digested with trypsin/Lys-C overnight at 37 °C. MS/MS spectra were obtained with a NanoElute ultra high-pressure Nano flow system (Bruker Daltonics, Billerica, MA, USA) as previously described [28]. For each condition, three independent experiments were carried out and sample tests were performed using Student’s t-test with a permutation-based FDR (False Discovery Rate) of 0.05.

### Electronic supplementary material

Below is the link to the electronic supplementary material.

Supplementary Material 1

Supplementary Material 2

Supplementary Material 3

## Data Availability

The LC-MS/MS proteomics data have been deposited to the Integrated Proteome Resources (iProX) with the dataset identifier IPX0007035001 (https://www.iprox.cn/page/home.html). All the genome sequences generated and analyzed during the current study are available in the NCBI repository (https://www.ncbi.nlm.nih.gov/bioproject/PRJNA1020763).

## Abbreviations

BHI: Brain Heart Infusion
TS: tryptone soya
MH: Mueller-Hinton
MIC: minimum inhibitory concentration
ROS: reactive oxygen species
RNS: reactive nitrogen species
