# Supplementary material for: Proteomic characterization of persisters in Enterococcus faecium
Source: BMC Microbiol. 2024 Jan 3;24:9. doi: 10.1186/s12866-023-03162-8 (PMC10765921; doi:10.1186/s12866-023-03162-8)
Supplement: Supplementary file 2 — Supplementary Material 2 [file 12866_2023_3162_MOESM2_ESM.docx]

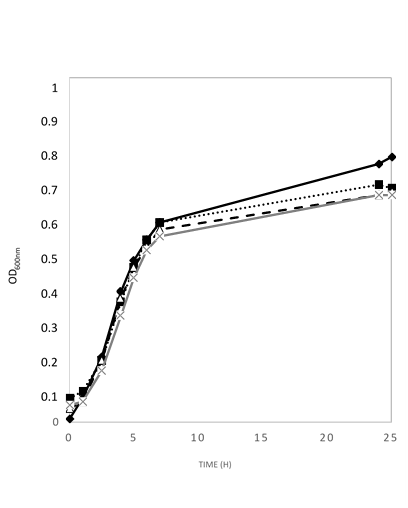


**Fig. S1 :** Growth curves of *E. faecium* AUS 0004 strain (black line) and three strains of persister cells (gray and dotted lines) in MH medium at 37°C.
