# Supplementary material for: Proteomic characterization of persisters in Enterococcus faecium
Source: BMC Microbiol. 2024 Jan 3;24:9. doi: 10.1186/s12866-023-03162-8 (PMC10765921; doi:10.1186/s12866-023-03162-8)
Supplement: Supplementary file 3 — Supplementary Material 3 [file 12866_2023_3162_MOESM3_ESM.docx]

**Fig. S2** Volcano plots of proteins differentially expressed in stationary phase and persister cells (red spots). A: comparison T_48h_ vs Expo. B comparison T_48hcip_ vs Expo.
